# Analytical Evaluation of Dried Blood Spot and Rapid Diagnostic Test as a New Strategy for Serological Community Screening for Chronic Chagas Disease

**DOI:** 10.3389/fcimb.2021.736630

**Published:** 2021-09-15

**Authors:** Aroa Silgado, Lídia Gual-Gonzalez, Adrián Sánchez-Montalvá, Inés Oliveira-Souto, Lidia Goterris, Nuria Serre-Delcor, Juliana Esperalba, Jordi Gomez-i-Prat, Candela Fernández-Naval, Israel Molina, Tomas Pumarola, Elena Sulleiro

**Affiliations:** ^1^Department of Microbiology, Vall d’Hebron University Hospital, Universitat Autònoma de Barcelona, PROSICS Barcelona, Barcelona, Spain; ^2^Laboratory of Vector-Borne and Zoonotic Diseases, Arnold School of Public Health, University of South Carolina, Columbia, SC, United States; ^3^Department of Infectious Diseases-Drassanes, Vall d’Hebron University Hospital, Universitat Autònoma de Barcelona, PROSICS Barcelona, Barcelona, Spain

**Keywords:** *Trypanosoma cruzi*, dried blood spot (DBS), rapid diagnostic test (RDT), serological screening, community strategies

## Abstract

**Background:**

Chagas disease is a public health problem not only in Latin America, but also in other regions, including Spain, due to migration movements. Conventional serological diagnosis requires an invasive sample (plasma or serum) and a well-equipped laboratory. To circumvent those limitations, blood samples dried on filter paper (DBS) or Rapid Diagnostic Test (RDT) could be a practical alternative to reference protocol for serological screening in epidemiological studies. We evaluated the usefulness of dried blood sampling and a rapid diagnostic test (Trypanosoma Detect™) for the detection of antibodies against *T. cruzi* for their use in community-based screening.

**Methodology/Principal Findings:**

A total of 162 stored paired whole-blood and serum samples from Latin American migrants and 25 negative-control blood samples were included. Diagnosis of chronic Chagas disease was performed in serum according to WHO algorithms. Blood samples were retrospectively collected as dried spots and then analyzed using two different serological techniques, enzyme-linked immunosorbent assay (ELISA) and electrochemiluminescence immunoassay (E-CLIA). Whole-blood samples were also used to evaluate a rapid diagnostic test based on immunochromatography. A better correlation with conventional serum was observed in dried blood elutes using E-CLIA than ELISA (97% vs. 77% sensitivity, respectively). Both assays reported 100% specificity. The median cut-off index values of E-CLIA for dried blood were significantly lower than those for serum (138.1 vs. 243.3, *P*<0.05). The Trypanosoma Detect™ test presented a sensitivity and specificity of 89.6% and 100%, respectively.

**Conclusions:**

The detection of antibodies against *T. cruzi* in dried blood samples shows a higher sensitivity when using E-CLIA compared with ELISA. Trypanosoma Detect™ is easier to use but has a lower sensitivity. Hence, we propose a sequential strategy based on performing the rapid test first, and a negative result will be confirmed by DBS-ECLIA for use in community Chagas disease screening programs.

## Introduction

Chagas disease (CD), a neglected tropical disease caused by the parasite *Trypanosoma cruzi*, is estimated to affect between 6 and 8 million people worldwide (World Health Organization, 2020). This vector-borne disease, endemic in Latin America, has changed its epidemiology due to population migrations out of the endemic area (Stanaway and Roth, 2015). Consequently, the number of reported cases of CD in European countries has increased in recent years, especially Spain (Requena-Méndez et al., 2015), which has the largest population of CD patients (Gómez i Prat et al., 2019).

Due to the psycho-emotional and socio-anthropological barriers that CD presents (Sanmartino et al., 2015), community screening is a fundamental tool to enhance the diagnosis of CD in order to offer integral management, including specific treatment. Despite improvements in diagnostic tools and screening programs for CD, there is still a large gap to cover in order to reach most of the susceptible population (Gómez i Prat et al., 2019).

The vast majority of CD patients living in non-endemic areas are in the chronic stage of the disease (Pérez-Molina et al., 2012). The diagnosis of this phase relies on the detection of IgG antibodies against *T. cruzi*. Serological assays, such as the indirect immunofluorescence assay, indirect haemagglutination, or enzyme-linked immunosorbent assay (ELISA) are commonly used (Rassi et al., 2012). The World Health Organization (WHO) guidelines recommend performing at least two different assays to diagnose the infection. All these serological assays present high sensitivity and specificity, nonetheless they are time-consuming, and technically demanding (Egüez et al., 2017). Furthermore, resources and personnel are required to perform the common serological tests (Egüez et al., 2017).

The use of serum/plasma limits the wider application to non-clinical settings, such as community screening programs (McDade et al., 2020). In order to circumvent this limitation, dried blood spots (DBS) from finger stick sampling has been explored. This type of sample is low cost, easy to collect, and simplifies the transport and storage of blood samples (Parker and Cubitt, 1999). Numerous uses for DBS, ranging from the diagnosis of infectious diseases to epigenetic studies, have been described elsewhere (McClendon-Weary et al., 2020).

For mass-screening surveys and intervention campaigns, a rapid, sensitive and easy-to-use diagnostic test would be valuable (Shah et al., 2014; Egüez et al., 2017). Currently, Rapid Diagnostic Tests (RDTs) have been developed for a range of tropical diseases, including *T. cruzi* infection (Reithinger et al., 2010). These are defined as equipment-free devices and are less technically demanding and time consuming than classic serological techniques (Angheben et al., 2016; Angheben et al., 2019). In addition, they provide results within minutes (Angheben et al., 2016). Remarkably, many of them can be performed on whole finger stick blood (Shah et al., 2014).

This study aims to evaluate the utility of DBS sampling and RDT in the detection of antibodies against *T. cruzi* for their application in community CD screening studies.

## Materials and Methods

### Study Population and Samples

A total of 162 whole blood stored samples were used retrospectively from Latin American migrants from endemic CD countries that had a previous diagnosis for chronic Chagas disease. *T. cruzi* infection status of the enrolled patients was established based on the consensus results of two conventional assays for IgG anti-*T. cruzi* (Pan American Health Organization, 2019): serum samples were tested by an electrochemiluminescence immunoassay (E-CLIA) (Elecsys Chagas, Roche Diagnostics, Manheim, Germany) and those with a positive result were subsequently analyzed using a commercial ELISA (Ortho *T. cruzi* ELISA, Johnson & Johnson, High Wycombe, United Kingdom). Infection was confirmed when the serum sample was positive for both assays.

Both blood and serum samples were stored at 4°C and were analyzed at the latest one week after collection.

Of 162 blood samples, 113 were used to obtain DBS; all were then analyzed by E-CLIA and 97 by ELISA after their reconstitution. For the RDT study, 91 of the 162 blood samples were used. Not all samples could be evaluated in all tests due to the availability of reagents and material available for the study.

**Figure 1:**
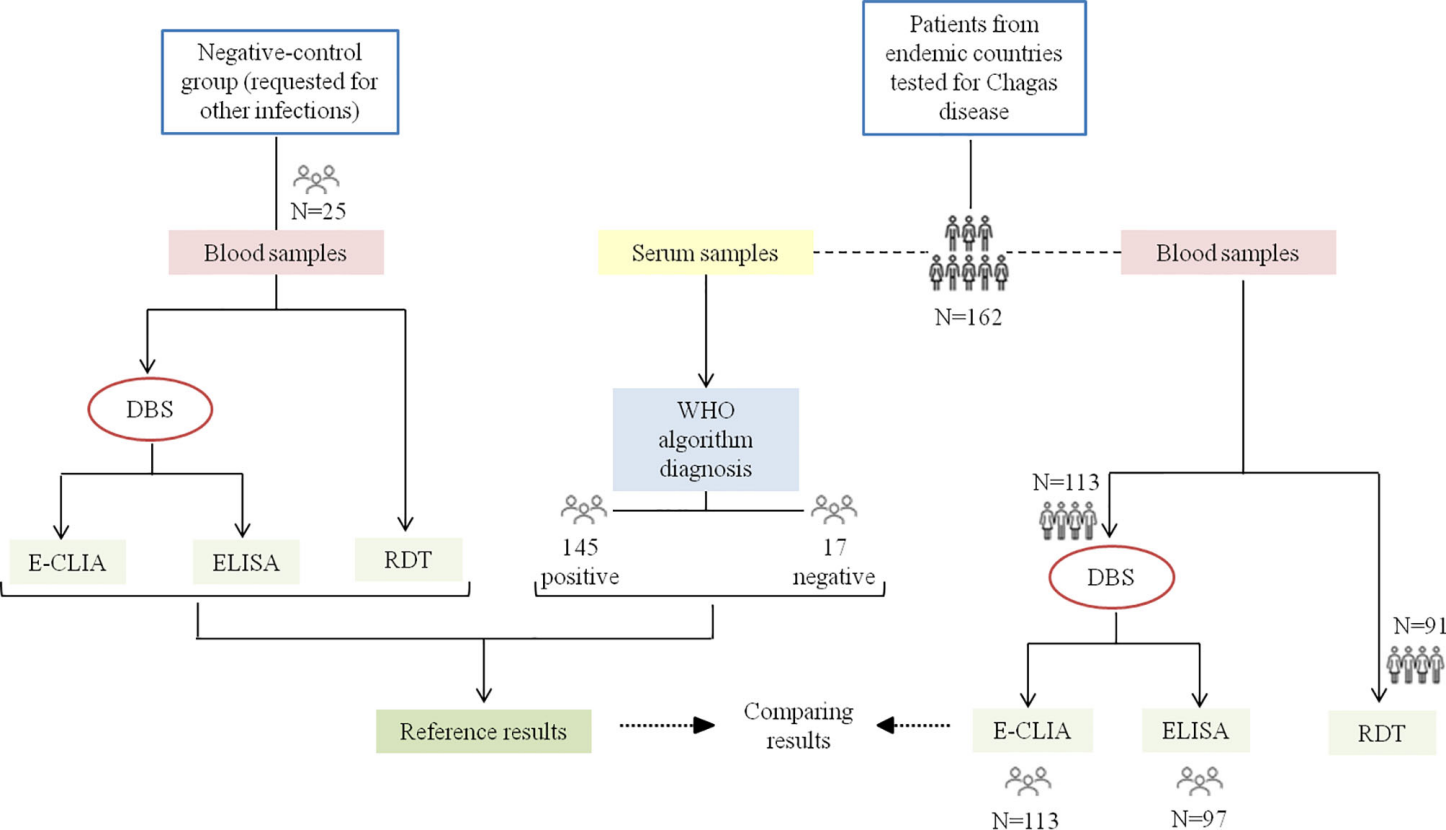
Algorithm of the samples used in this study. From patients tested for CD, serum samples were first analyzed by E-CLIA and those with a positive result by ELISA (WHO algorithm diagnosis). The blood samples were used to obtain DBS and to evaluate the RDT. The reference results are those obtained in the serum samples, as well as in the blood samples of the negative control group (serologically negative for *T. cruzi*). The number of samples tested in each technique is specified.

Twenty-five blood samples from patients with no travel history to *T. cruzi* endemic areas (samples to rule out other infections, i.e., Cytomegalovirus or BK virus), were used as the negative-control group (**Figure 1**).

Fifty µL of whole blood were used to fill one 8-mm-diameter circle on filter-paper cards (Whatman 903™ Specimen Collection Paper; GE Healthcare Ltd, Cardiff, United Kingdom). This procedure was repeated to fill two circles. Each card was dried overnight at room temperature (approximately 25°C) and the elution of DBS was carried out the day after drying. For their reconstitution, both two paper circles were placed in 300 µL of phosphate-buffer saline (PBS) and incubated overnight with gentle rotation at room temperature (Holguín et al., 2013). The Whatman paper was then carefully removed and the eluted blood spots were centrifuged for 2 minutes at 12500 rpm to clear away any remaining paper from the supernatant and stored at 4°C until analysis (within 1-2 days after reconstitution).

### Detection of IgG Anti-*T. cruzi* in DBS

The presence of IgG antibodies against *T. cruzi* was determined in the eluted DBS samples using two different techniques:

(1) Elecsys Chagas (Roche Diagnostics), an automated E-CLIA for the qualitative determination of antibodies to *T. cruzi*, following the manufacturer’s instructions and analyzed with the automated COBAS 8000 analyzer (Roche Diagnostics).

(2) Chagas ELISA IgG+IgM (Vircell Microbiologists, Granada, Spain), following the manufacturer’s instructions and using automated equipment, the DS2 ELISA Processor (Dynex Technologies, Chantilly, VA).

Results for both serum and DBS were interpreted according to the Elecsys Chagas assay manufacturer’s cut-off value: samples with cut-off index (COI) values of ≥1.0 were considered reactive, while <1.0 were labeled as non-reactive. For the Chagas ELISA IgG+IgM system, DBS samples were considered positive if the antibody index (sample optical density/cut-off serum mean optical density) was above 1.1, grey area if antibody index was 0.9-1.1, and negative if antibody index was below 0.9.

### Rapid Diagnostic Test

The Trypanosoma Detect™ Rapid Test (InBios International, Inc., Seattle, WA) was also evaluated. A total of 20 µL of whole blood was used and processed according to the manufacturer’s instructions.

### Statistical Analysis

The relative sensitivity (S; true positive/total positive) and relative specificity (E; true negative/total negative) from each test were calculated. Qualitative variables were expressed as absolute frequencies and percentages, and quantitative variables as the median and interquartile range (IQR). Continuous variables were compared using the *t*-test or Mann-Whitney U test when appropriate. The Cohen Kappa coefficient was used to analyze the level of agreement between tests. An appropriate cut-off value for the DBS samples in the Elecsys Chagas (E-CLIA) assay was determined using a receiver operating characteristic (ROC) curve. The Youden index was calculated, which is a measure of the overall discriminative power of a diagnostic procedure.

Statistical analyses were carried out using the R Studio software version 3.5.3 (R Development Core Team, Vienna, Austria).

### Ethics Statement

This study is based on retrospective stored samples from Latin American patients attended at the Hospital Universitari Vall d’Hebrón (HUVH). Review Board approvals (PR(SC)253/2012) were obtained from the Ethics Committee of the Vall d’Hebron Research Institute, according to the principles expressed in the Declaration of Helsinki. All samples were anonymized before being analyzed.

## Results

A total of 162 blood samples from 145/162 (89.5%) patients positive for *T. cruzi* infection and 17/162 (10.5%) negative for *T. cruzi* infection were evaluated in this study.

**Figure 2:**
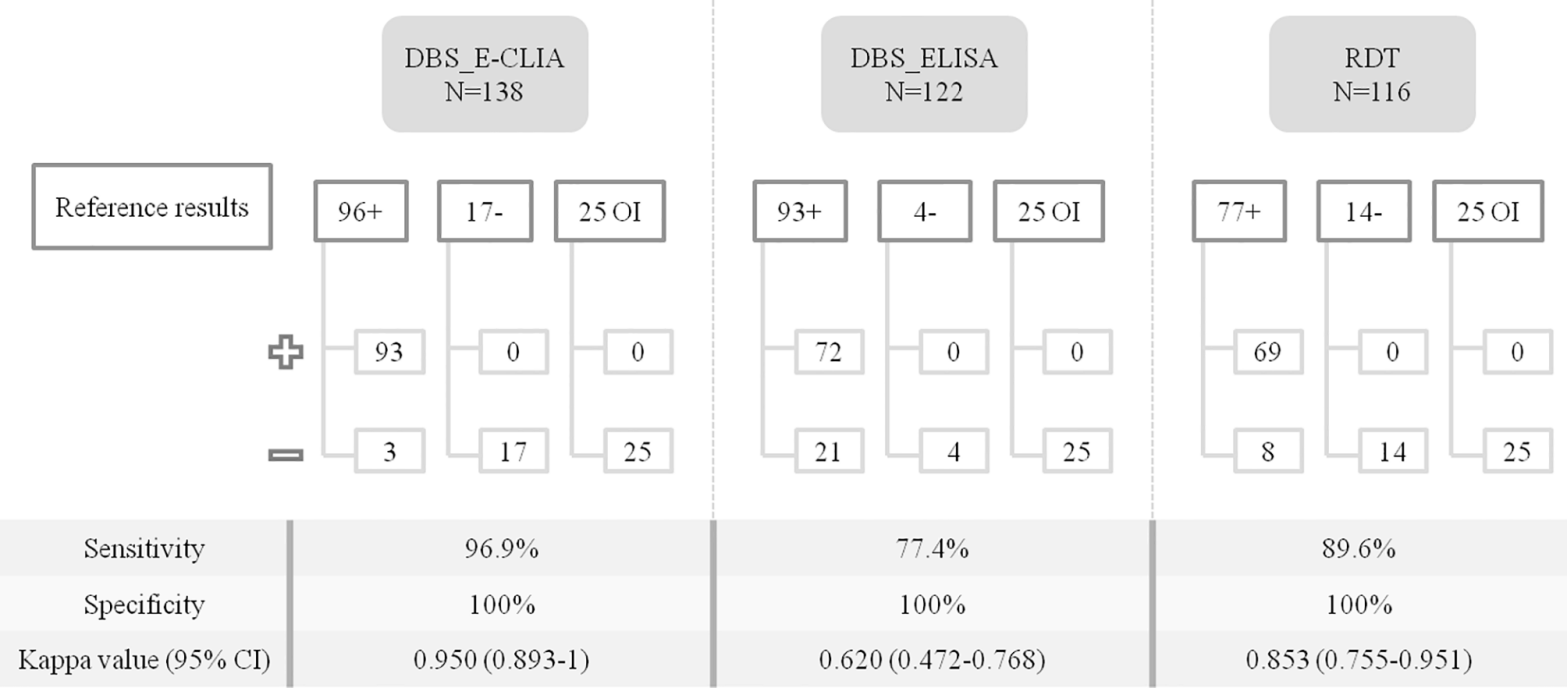
Comparison of *T. cruzi* diagnostic performance on DBS samples and RDT. The number of samples tested with each methodology is specified, also whether they were positive or negative and whether they were samples from patients with other infections (serologically negative for *T. cruzi*). +, samples testing positive for IgG anti-*T. cruzi*; -, samples testing negative for IgG anti-*T. cruzi*; OI, other infections: samples from individuals tested for other infections; 95% CI, 95% confidence interval.

Overall, **Figure 2** summarizes the samples tested with each methodology and the results obtained from the comparison with the reference results.

**Figure 3:**
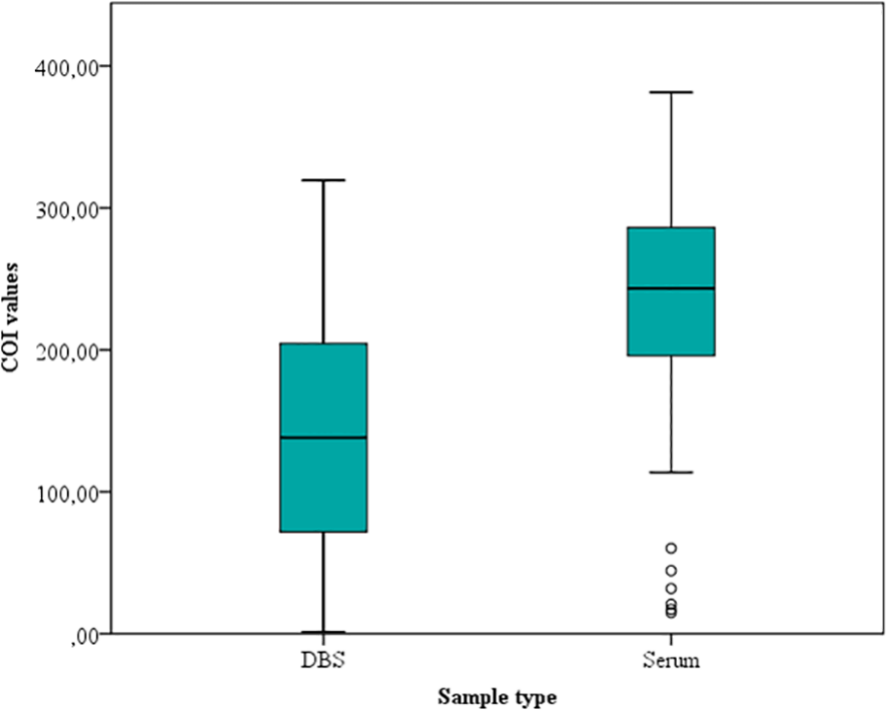
Dried Blood Spot and serum COI values obtained by the Elecsys Chagas assay.

DBS E-CLIA shows the best agreement, achieving an S of 96.9% [95% confidence interval (CI), 90.5 to 99.2]. Serum COI values were higher than those obtained with DBS; the median value for serum was 243.3 (IQR, 195.9 to 286.3), while the median value for DBS was 138.1 (71.7 to 204.4) (p-value <0.05). The distribution of the COI values for both methodologies is shown in **Figure 3**.

Compared with serum, 3/138 (2.2%) DBS samples were inappropriately classified as negative. COI values in the respective serum sample indicated that these three samples had low IgG anti-*T. cruzi* levels [DBS median, 0.5 (0.3 to 0.6) vs. serum median, 9.7 (7 to 10.8)].

**Figure 4:**
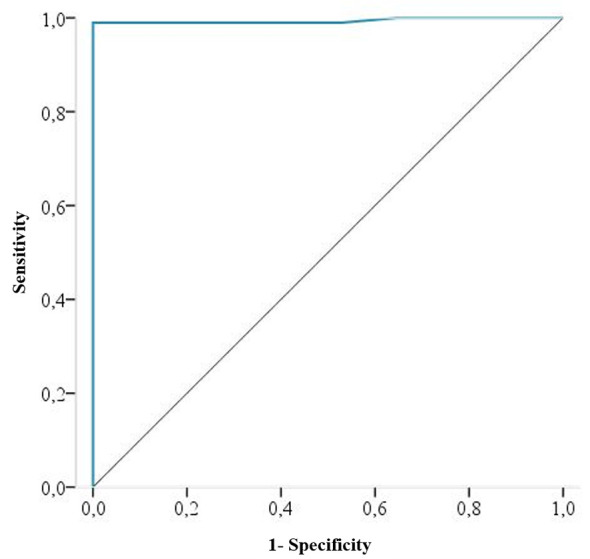
ROC curve for DBS results considering serum samples as the reference method. The ROC curve area is 0.994 (95% IC, 0.981 to 1).

Taking serum sample results as the reference method, the S and E for all potential COI values for the DBS samples in E-CLIA were obtained by means of a ROC curve (**Figure 4**).

The performance of E-CLIA on DBS samples improves when the cut-off threshold changes from ≥1 to ≥0.31, achieving an S and E of 99% and 100%, respectively. Correspondingly, the false-negative samples decreased from three to one.

The lowest Cohen Kappa coefficient was found between DBS-ELISA and reference results, which had the highest number of samples with discordant results. In particular, 21/122 (17.2%) samples with a false-negative result were reported, lowering the S to 77.4% (95% CI, 67.4 to 85.2%). All these 21 DBS samples were positive when analyzed by E-CLIA (in both DBS and serum samples).

As regards RDT, 8/77 (10.4%) E-CLIA serum positive samples were misclassified as negative, reaching an S of 89.6% (95% CI, 80 to 95.1). In this group of false-negatives, the median COI value in paired serum samples was 16.1 (IQR, 11.3 to 43.1), while the median value in serum samples for the true-positive group was 202.9 (IQR, 83.5 to 244.4), with a statistically significant difference (p-value <0.001).

In all techniques, the E was 100% (95% CI, 89.7 to 99.8 for E-CLIA, 85.4 to 99.7 for ELISA, and 88.8 to 99.8 for RDT).

## Discussion

CD is a potentially life-threatening illness with a high percentage of un-diagnosed cases in non-endemic countries as Spain (Basile et al., 2011; World Health Organization, 2020).

Therefore, it is essential to improve access to CD diagnosis and treatment and community-based testing would be a good strategy (Gómez i Prat et al., 2020). In this study, we evaluated two diagnostic procedures that could be applied in serological screening programs: sample collection through DBS and an RDT.

DBS sampling offers a good alternative to conventional serum samples, for those situations where there are no facilities or expertise to properly obtain, transport and store blood specimens (Tuaillon et al., 2020). Blood collection on DBS can be performed using a finger stick in adults and older children or a heel-prick in neonates and infants, making it more suitable for screening programs (Kania et al., 2013).

In the present study, DBS sampling analyzed by the E-CLIA technique the results obtained were comparable to conventional serum samples, reaching a sensitivity and specificity of 96.9% and 100%, respectively. A similar sensitivity was reported in the study of Holguín et al. (2013). However, they reported a lower specificity, which may be due to the fact that they tested a greater number of negative samples.

The amount of reconstituted elute from filter paper can vary depending on several factors, such as blood volume, collection and reconstitution protocols, as well as the sample storage (Rodríguez-Pérez et al., 1999; McClendon-Weary et al., 2020). In our study, lower COI values were reported for DBS samples than for serum samples (median of 138.1 vs. 243.30). Moreover, we detected 3 false-negatives (two of them also negative with the RDT); probably due to the lower antibody titres in DBS samples than in serum due to sample dilution. Recalculating the cut-off value would be useful to determine an optimal antibody recovery protocol and improve detection in patients with low antibody titres (Holguín et al., 2013). The new cut-off determined for DBS samples was much lower than for serum samples (≥0.31 vs. ≥1, respectively), with the consequent risk of leading to an erroneous interpretation. Therefore, a future study including a larger number of samples would be necessary to accurately determine a cut-off value for DBS samples analyzed by Elecsys Chagas.

With ELISA, the sensitivity drops to 77.4%, with a high number of false-negative results (21 out of 122 samples). The most likely reason for this low correspondence with serum diagnosis could be that the paper scraps left behind could interfere with the correct reading of the samples. Unfortunately, due to the small volume of the samples, the analyses of discrepant samples could not be repeated. Higher sensitivity is also observed in serum samples when comparing with the results obtained by E-CLIA with ELISA (Antinori et al., 2018).

Differences in the analytical performance of DBS when analyzed by two different techniques (E-CLIA and ELISA) were found. Thus, a previous evaluation of DBS samples would be suitable to determine the accuracy of the diagnostic technique which will be used.

In recent years, the use of rapid tests to diagnose infectious disease has increased, including CD. The RDTs provide rapid, reliable, and accurate results if the test used is sensitive and specific (Sánchez-Camargo et al., 2014).

In our study, the performance data (S: 89.6% and E: 100%) obtained was close to the data reported in other studies using the same rapid test (Brutus et al., 2008; Lorca et al., 2008; Bern et al., 2009; Reithinger et al., 2010; Sánchez-Camargo et al., 2014). Other studies, using an improved version of this test (Shah et al., 2014), have also reported high sensitivity (Shah et al., 2014; Egüez et al., 2017). Interestingly, they used whole blood from a finger stick, as would be the idea in screening programmes.

RDTs are recommended for screening and surveillance in both endemic and non-endemic areas (Sánchez-Camargo et al., 2014). In non-endemic countries, with a low prevalence, the choice of using RDT for the screening of individuals should be followed by confirmation of the results in a reference laboratory (Sánchez-Camargo et al., 2014; Angheben et al., 2016).

A strategy based on a single RDT would be advantageous in terms of accessibility thus facilitating its implementation at the community level, but the sensitivity of RDT reflected in our study (89%) was not sufficient. Based on the results obtained in this study, the proposed strategy is based on the combined use of the rapid test with the subsequent confirmation of negative cases by means of the corresponding DBS sample analyzed with a more sensitive technique, such as E-CLIA, to reach a sensitivity of 97%. However, the addition of the DBS sampling involves restructuring the field equipment in order to be able to carry out the tests in the shortest possible time.

It is important to apply more effective strategies to strengthen the capacity of health systems to detect CD cases. Some endemic areas are initiating strategies to enhance access to diagnosis and therefore working on early treatment. However, in non-endemic areas, where access to diagnosis is not a problem, the main challenge is the lack of information from health professionals with policymakers about the need for screening and specific guidelines (Alonso-Padilla et al., 2019). Therefore, it is essential to know the true prevalence of *T. cruzi* among Latin American migrants in order to design an efficient screening strategy (Da Costa-Demaurex et al., 2019). Community-based activities allow us to get closer to the population at risk and are necessary to overcome psycho-emotional and socio-anthropological barriers. At the same time, they offer an on-site diagnosis, which has been shown to be very effective in other studies conducted in Europe (Navarro et al., 2011; Repetto et al., 2015; Navarro et al., 2017; Da Costa-Demaurex et al., 2019; Gómez i Prat et al., 2020).

Our study does, however, have limitations due to its retrospective nature. For example, the study lacks information regarding epidemiological and clinical data of the enrolled patients. Additionally, the DBS sampling and RDT were not performed on finger stick samples, as would be the idea in a community screening programme. Therefore, a field community study using whole blood from finger sticks would be interesting to confirm the present data and to evaluate these options in the right context.

To conclude, the use of DBS plus E-CLIA (Elecsys Chagas) could be a good option for the detection of IgG anti-*T. cruzi*, with an analytical performance similar to that obtained with conventional serology. Nevertheless, DBS specimens should be evaluated in the diagnostic technique to be used. Moreover, it would be desirable to evaluate an optimum cut-off value of Elecsys Chagas for DBS before processing this type of sample. The RDT (Trypanosoma Detect™) rendered a good performance for the rapid diagnosis of CD, with adequate sensitivity and specificity, but not sufficient to be used as the sole test in screening community programmes. Therefore, based in our results, we propose a sequential strategy based on, first, performing the easy-to-use rapid test and, second, confirming the negatives with a more sensitive technique, DBS E-CLIA, for implementation under field conditions or screening programs outside of health facilities. However, considering the limitations presented in this study, a future trial would be necessary to test such a strategy.

## Data Availability Statement

The original contributions presented in the study are included in the article/supplementary material. Further inquiries can be directed to the corresponding author.

## Ethics Statement

The studies involving human participants were reviewed and approved by Vall d’Hebron Research Institute. Written informed consent for participation was not required for this study in accordance with the national legislation and the institutional requirements.

## Author Contributions

Conceptualization: ES. Methodology: AS and LG-G. Formal analysis: AS and LG-G. Data curation & Investigation: AS, LG-G, AS-M, IO-S, LG, NS-D, JE, JG-i-P, CF-N, IM, TP, and ES. Writing-Original draft: AS and LG-G. Writing-Review & Editing: AS, LG-G, AS-M, IO-S, LG, NS-D, JE, JG-i-P, CF-N, IM, TP, and ES. Funding acquisition: IO-S and AS-M. All authors contributed to the article and approved the submitted version.

## Funding

This work has been supported by the Fundació la Marató TV3 (project number 20182610).

## Conflict of Interest

The authors declare that the research was conducted in the absence of any commercial or financial relationships that could be construed as a potential conflict of interest.

## Publisher’s Note

All claims expressed in this article are solely those of the authors and do not necessarily represent those of their affiliated organizations, or those of the publisher, the editors and the reviewers. Any product that may be evaluated in this article, or claim that may be made by its manufacturer, is not guaranteed or endorsed by the publisher.
